# Clinical and biochemical footprints of inherited metabolic diseases. XIV. Metabolic kidney diseases

**DOI:** 10.1016/j.ymgme.2023.107683

**Published:** 2023-08-12

**Authors:** Anke Schumann, Ulla T. Schultheiss, Carlos R. Ferreira, Nenad Blau

**Affiliations:** a Department of General Paediatrics, Adolescent Medicine and Neonatology, Medical Center-University of Freiburg, Faculty of Medicine, Freiburg, Germany; b Department of Medicine IV, Nephrology and Primary Care, Faculty of Medicine, and Medical Center, University of Freiburg, Institute of Genetic Epidemiology, Freiburg, Germany; c National Human Genome Research Institute, National Institutes of Health, Bethesda, USA; d Division of Metabolism, University Children’s Hospital, Zürich, Switzerland

**Keywords:** Inborn errors of metabolism, Inherited metabolic diseases, Tubulointerstitial nephritis, Kidney cysts, Chronic kidney disease, IEMbase

## Abstract

Kidney disease is a global health burden with high morbidity and mortality. Causes of kidney disease are numerous, extending from common disease groups like diabetes and arterial hypertension to rare conditions including inherited metabolic diseases (IMDs). Given its unique anatomy and function, the kidney is a target organ in about 10% of known IMDs, emphasizing the relevant contribution of IMDs to kidney disease. The pattern of injury affects all segments of the nephron including glomerular disease, proximal and distal tubular damage, kidney cyst formation, built-up of nephrocalcinosis and stones as well as severe malformations. We revised and updated the list of known metabolic etiologies associated with kidney involvement and found 190 relevant IMDs. This represents the 14th of a series of educational articles providing a comprehensive and revised list of metabolic differential diagnoses according to system involvement.

## 1. Introduction

This is the 14th in a series of articles that provide a comprehensive list of inherited metabolic diseases (IMDs) associated with specific signs and symptoms. The previous issues highlighted movement disorders [1], metabolic liver diseases [2], psychiatric presentations [3], metabolic cardiovascular diseases [4], cerebral palsy phenotype [5], metabolic dermatoses [6], ocular phenotype [7], neoplasias [8], metabolic ear disorders [9], metabolic myopathies [10], gastrointestinal symptoms [11] immunological defects [12], and respiratory manifestations [13]. This article is dedicated to IMDs with a kidney phenotype leading to chronic kidney disease (CKD).

## 2. Materials and methods

Source of the information was IEMbase, a knowledgebase of IMDs (http://www.iembase.org) [14]. As of July 13, 2023, IEMbase tabulates 1879 IMDs and 4107 corresponding clinical and biochemical signs and symptoms which have been grouped into 22 organ systems and conditions (Autonomic system, Cardiovascular, Dental, Dermatological, Digestive, Dysmorphic, Ear, Endocrine, Eye, Genitourinary, Hair, Hematological, Immunological, Metabolic, Muscular, Neurologic, Psychiatric, Kidney, Respiratory, Skeletal, Tumoral and Other). The clinical symptoms associated with kidney involvement (*n* = 68) were extracted from the ‘Kidney’ group. The nosology of IMDs [15] was reclassified according to the International Classification of Inherited Metabolic Disorders, ICIMD [16].

## 3. Kidney disease in inborn errors of metabolism

The ICIMD includes 1450 disorders and is constantly growing [16]. Of these, 190 IMDs have been associated with kidney disease of different types to date, approximating 10% of all IMDs. Of note, CKD of more common causes (arterial hypertension, diabetes mellitus, glomerulonephritis) has a prevalence of 10–15% in adults as well, which underlines the relevance of CKD in IMD. Regardless of origin, CKD is associated with high morbidity and mortality [17]. CKD can be defined as abnormalities of kidney structure or function shown by a glomerular filtration rate of <60 ml/min per 1.73m^2^ in adults and <90 ml/min per 1.73m^2^ in children or markers of kidney damage or a combination of both [18]. These kidney abnormalities need to persist for at least 3 months [19]. The final consequence is kidney failure (KF).

**Fig. 1.**
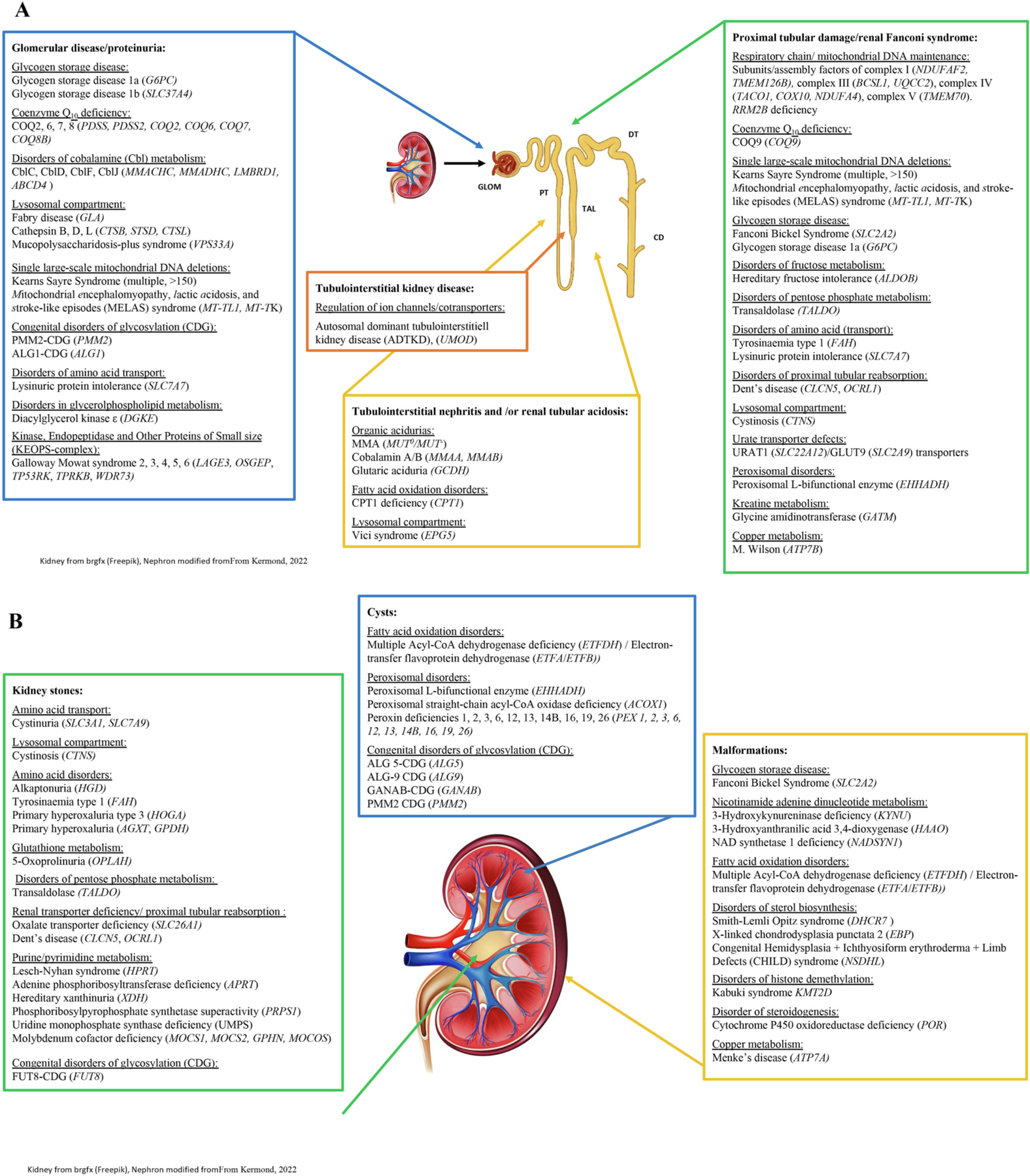
Fig. 1A depicts the segmental distribution of inherited metabolic diseases (IMD) affecting kidney function. The respective IMDs are clustered along the nephron; glomerulus (GLOM, blue), proximal tubule (PT, green), thick ascending limb (TAL, orange), distal convoluted tubule (DCT, yellow) and collecting duct (CD). Genes in italic. Fig. 1B shows IMDs leading to precipitations/stone formations (green), cysts (blue) or kidney malformations/structural abnormalities (yellow). Genes in italic. (For interpretation of the references to colour in this figure legend, the reader is referred to the web version of this article.)

Markers of kidney damage include albuminuria (albumin:creatinine ratio ≥ 30 mg/g), urinary sediment abnormalities, abnormalities due to tubular disorders, histopathological findings, structural abnormalities and/or a history of kidney transplantation [18]. IMDs basically affect all parts of the nephron (the functional unit of the kidney starting at the glomerulus and ending with the collecting duct), kidney interstitial space and the draining urinary tract (Figs. 1A and B).

## 4. Glomerular disease

Glomerular disease is a common feature in IMD. The glomerulus has a central role for a normal kidney function: it is the starting point of the nephron, the functional unit of the kidney, where the primary urine is filtered at a rate of ~120–140 ml/min in adults [20]. Due to their complex architecture (vasa afferens/efferens, fenestrated vessels, podocytes, Bowman capsule, glomerular basal membrane, mesangial cells) glomeruli are the target of numerous harmful processes like immunological, metabolic, vascular, and malignant disorders [21]. Proteinuria is a frequent finding in glomerular disease. However, proteinuria has to be differentiated into glomerular loss across the glomerular filtration barrier (mainly albumin) and tubular loss caused by reabsorption deficiency in the proximal tubule (PT, minor fraction is albumin) [22].

Nephrotic syndrome is caused by podocyte damage and leads to nephrotic-range proteinuria (protein excretion rate PER >3500 mg/d or protein creatinine ratio PCR > 3500 mg/g), hypoalbuminemia, hyperlipidemia, and oedema. Due to its etiology, histopathological patterns can be very different (e.g., immune complex deposits, mesangial proliferation, etc.).

Fabry disease (FD) is caused by deficiency of the lysosomal enzyme alpha-galactosidase A due to variants in *GLA*. The prevalence of kidney disease in FD is as high as 55%. The earliest kidney related sign is proteinuria and decline of the glomerular filtration rate. Parapelvic cysts found in 43% of all FD patients are another frequent and disease-characteristic sign affecting the kidney. Podocyturia can be frequently found [23]. Lysosomal protease cathepsins belong to the same compartment and are involved in kidney damage. >15 cathepsins have been identified and clustered in 3 different groups based on their catalytic active sites: serine (cathepsin A, G), aspartate (cathepsins D and E), or cysteine (cathepsins B, C, F, H, K, L, O, S, V, X, and W). Especially alterations in cathepsins B, D and L have been associated with glomerulosclerosis and glomerular kidney disease [24].

Glycogen storage disease type 1 (GSD1) is one of the most common glycogen storage diseases and is caused by deficient glucose-6-phosphate metabolism. Two subtypes, GSD 1a, caused by variants in the *G6PC* gene and GSD 1b, due to pathogenic *SLC37A4* variants are known. Kidney involvement primarily manifests with microalbuminuria and thus glomerular involvement. Early and recent studies (*n* = 26, GSD1a) [25] and [*n* = 32; 26 GSD1a, 6 GSD1b] [26] report around 31% of patients with microalbuminuria (abnormal increased excretion rate of albumin in the urine ranging from 30 to 299 mg/g creatinine [27]), mostly associated with hyperfiltration (increased GFR above normal values seen e.g. in early phases of kidney disease [28]). Dietary modifications and angiotensin converting enzyme (ACE) inhibitors seem to have a positive effect in stabilizing kidney function. Nephrocalcinosis and/or nephrolithiasis (around 19% of the study populations) have also been reported concerning kidney involvement in GSD 1. Tubular dysfunction has been ascribed to glycogen accumulation and is less frequent. In a small cohort of nine patients, six were unable to sufficiently secret hydrogen ions after bicarbonate load [29].

Mitochondrial diseases are frequently associated with glomerular involvement: Defects in the biosynthesis of coenzyme Q_10_ deficiency have been associated with glomerular disease. To date, about 144 patients from 95 families have been identified. Genes involved comprise *PDSS, PDSS2, COQ2, COQ6, COQ7* and *COQ8B* (previously termed *ADCK4*) [30]. Patients predominantly present with severe nephrotic syndrome. Early identification is crucial since the disease is treatable by coenzyme Q_10_ supplementation.

Mitochondrial encephalomyopathy, lactic acidosis, and stroke-like episodes (MELAS) syndrome is a mitochondrial multisystem disorder predominantly caused by pathogenic variants in the *MT-TL1*, but also associated with *MT-TK* variants. Kidney involvement occurs in <25% of the cases but can present with severe focal segmental glomerulosclerosis [31]. In a patient cohort with single large-scale mitochondrial DNA (SLSMD) deletions, 85% of the patients showed signs of glomerular and tubular dysfunction [32]. Also, more common mitochondrial depletion syndromes like Kearns-Sayre and Pearson syndromes have been associated with both glomerular and tubular phenotypes [25,27].

The frequency of steroid resistant nephrotic syndrome (SRNS) varies with age. The genetic causes of SRNS are numerous. Among them, different types of ultra-rare Galloway-Mowat syndrome, a multisystem disease with a predominant kidney phenotype, lead to SRNS (e.g., caused by pathogenic variants in *LAGE3*), *OSGEP*, *TP53RK*, *TPRKB*, or *WDR73*) [33]. Pathogenic variants in components of the nuclear pore complex of podocytes, like *NUP133* and *NUP107*, have also been associated with SRNS [34].

Congenital disorders of glycosylation (CDG) are caused by defects in the assembly and processing of oligosaccharides in glycoproteins or glycolipids, leading to multisystem disease. Coagulation abnormalities, liver involvement, ataxia and developmental delay are leading features, while the presence of seizures is variable. Phosphomannomutase 2 (PMM2) deficiency is the most frequent N-linked glycosylation defect. In a cohort of 933 PMM2 patients, 6% suffered from kidney abnormalities, most of them present at birth. Proteinuria and cysts were the most common findings; however, 10% of patients with kidney disease suffered from nephrotic syndrome [35]. Deficiency of the β−1,4 mannosyltransferase caused by pathogenic *ALG1* variants lead to ALG1-CDG. Around 20% of ALG1-CDG patients suffer from congenital nephrotic syndrome and CKD. These associations point to the glomerulus as the primary target in CDG syndromes. Mucopolysaccharidosisplus syndrome caused by variants in the *VPS33A* gene is an ultra-rare condition resembling the clinical picture of mucopolysaccharidosis with some additional features like nephrotic syndrome and platelet dysfunction [36].

Hemolytic uremic syndrome (HUS) is defined as anemia, acute kidney injury due to blood clotting in the glomeruli and low platelets. It is usually caused by infectious agents (e.g., *E. coli*, classical HUS), but can also be attributed to monogenic diseases which are primarily impacting cobalamin and folate metabolism (atypical HUS, aHUS): Combined methylmalonic acidemia and homocystinuria defects (CblC, CblD, CblF, CblJ) do present with HUS in 10–25% of reported patients (*n* = 396) [37]. Furthermore, disorders in glycerophospholipid metabolism (variants in diacylglycerol kinase ε; *DGKE*) are known as a cause of aHUS in pediatric patients. The interplay between diacylglycerol kinase ε and the complement system is a presumed pathomechanism [38].

## 5. Tubulopathy

Kidney tubular epithelial cells are responsive for passive and active transport processes of fluids and electrolytes and control acid base homeostasis [39]. Quite a number of these processes are ATP-dependent. Disruption of this process results in tubulopathy [39]. The term tubulopathy is rather broad; first, kidney tubular segments can be divided into four main segments: proximal tubule (PT, reabsorption of the majority of solutes and water), thick ascending loop of Henle (TAL, concentration and reabsorption of sodium), distal convoluted tubule (DCT) and collecting duct (CD, sodium and water reabsorption in dependency of aldosterone stimuli) [39].

Renal Fanconi syndrome (RFS) affects the PT and is characterized by generalized aminoaciduria, glucosuria, phosphaturia and metabolic acidosis [40]. RFS is a known feature of IMDs of different origin. Due to disturbances in energy homeostasis, mitochondrial disorders quite frequently present with tubulopathies and RFS in particular [41]. Deficiencies of different respiratory chain complexes and their assembly factors have been associated with RFS: subunits and assembly factors of complex I (*NDUFAF2* [30] and *TMEM126B* [42]), complex III (*BCSL1* [43] and *UQCC2*), complex IV (*TACO1, COX10, NDUFA4*), and complex V (*TMEM70*) [41] have been reported. *RRM2B* deficiency belongs to the group of mitochondrial DNA maintenance defects. Its severe encephalomyopathic form presents with PT tubulopathy in humans. *RRM2B* gene deletion in a mouse model led to fragmented, degenerated mitochondria in murine PT cells and a decline of antioxidant capacities [44]. Disorders of Coenzyme Q_10_ deficiency have been mainly related to glomerulopathy (see section glomerulopathies). Only rare cases of coenzyme biosynthesis protein 9 (*COQ9)* have been linked to renal tubular dysfunction [45,46]. RFS has been reported in patients with mitochondrial deletion syndrome (Kearns-Sayre syndrome, Pearson syndrome) and with monogenic diseases of mitochondrial DNA deficiency like MELAS syndrome, heavy strand promoter (HSP) variants of mitochondrial DNA, and tRNA^Phe^ variants [30].

Pyruvate carboxylase (PC) synthesizes oxaloacetate from pyruvate and is key for gluconeogenesis from lactate or alanine. PC deficiency has been reported to induce RFS; however, RFS is not the predominant problem in PC patients [47,48].

Disturbances in carbohydrate metabolism affect the PT as well: Hereditary fructose intolerance caused by pathogenic variants in the *ALDOB* gene leads to accumulation of toxic fructose-1 phosphate and to intracellular depletion of inorganic phosphate and ATP, both of which are responsible for PT dysfunction [49]. Carbohydrate transmembrane transport is deficient in Fanconi-Bickel syndrome (FBS) caused by variants in the *SLC2A2* gene [50,51]. Glycogen storage has been related to RFS and renal tubular acidosis [50] in FBS.

Transaldolase coded by the *TALDO* gene is an enzyme of the pentose phosphate pathway. CKD is a predominant feature of this rare condition mainly presenting with a proximal tubular dysfunction (aminoaciduria, proteinuria) in about 25% of patients. Distal tubular dysfunction (loss of electrolytes) has also been reported [52,53].

Dent’s disease, caused by variants in the *CLCN5* (Dent disease 1) or *OCRL1* (Dent disease 2) genes, leads to proximal tubular dysfunction in basically all affected patients. While *CLCN5* codes for the CLC family of Cl^−^ channels/transporters, *OCRL1* codes for a phosphatidylinositol bisphosphate (PIP_2_) 5-phosphatase involved in endocytotic uptake processes [54].

PT damage is also found in disturbed transport of amino acids and amino acid disorders: RFS is characteristic for patients suffering from cystinosis caused by variants in the *CTNS*. Infantile nephropathic cystinosis is the most common hereditary cause of RFS in children and occurs within the first year of life [55]. Lysinuric protein intolerance is caused by variants in the *SLC7A7*, which codes for a cationic amino-acid transporter. Tubulopathy was reported in all patients of the cohort (*n* = 11) while proteinuria (25%) and CKD (43%) was observed in fewer cases [56]. Rare glomerular involvement can be very heterogeneous ranging from membranoproliferative glomerulonephritis to glomerular amyloidosis [57].

Tyrosinemia type 1 [58] is another disease associated with RFS. Nitisinone treatment and a tyrosine-reduced diet can reverse the kidney phenotype [59].

Wilson disease, a disorder of copper metabolism where copper cannot be properly disposed of, can present with a variable kidney phenotype including RFS, proteinuria and nephrolithiasis. Although the tubulopathy is intrinsic to the disease, the proteinuria is likely a consequence of of D-Penicillamine treatment [60].

Another form of inherited RFS caused by mistargeting of the peroxisomal L-bifunctional enzyme (Enoyl-CoA hydratase +3-Hydroxyacyl CoA dehydrogenase; *EHHADH*), which is responsible for peroxisomal fatty acid oxidation in PT cells, underlines the importance for mitochondria in the PT [61].

Furthermore, deficiencies in urate handling in the PT have been associated with exercise-induced acute kidney injury with acute tubular necrosis: Loss of function of the apical URAT1 (*SLC22A12*) and basolateral GLUT9 (*SLC2A9*) transporters lead to kidney hypouricemia which can be aggravated by physical stress [62].

In summary, RFS is caused by IMDs predominantly associated with energy production or energy supply covering a broad spectrum of energy dependent transport processes, substrate deficiency or deficient mitochondrial function.

However, IMDs have also been associated with tubular disease not affecting primarily the PT or having a particular “tubular” phenotype with salt loss or aminoaciduria, but with tubular abnormalities at a subcellular level or tubulointerstitial nephritis. Moving down the nephron, pathogenic variants in the *UMOD* gene expressed in the TAL lead to autosomal dominant tubulointerstitial kidney disease (ADTKD) and have an overall prevalence of 2% in patients with CKD. While 84% of affected individuals have CKD, 43% progress to KF, highlighting this monogenic defect as one of the most frequent and severe causes of IMDs with a kidney phenotype [63].

Methylmalonic (MMA) and propionic aciduria (PA) belong to the group of organic acidurias. Both diseases have been associated with tubulointerstitial nephritis and/or renal tubular acidosis. Kidney disease is strongly correlated with the subtype: Patients suffering from the severe form of methylmalonyl-CoA mutase (MUT) deficiency (*MUT*^0^) showing low residual enzyme activity and high levels of methylmalonic acid, have an earlier and fast progressing kidney phenotype with reduced glomerular filtration rate, while *MUT*^−^ patients (residual enzymatic activity above 10%) and patients suffering from MMA caused by cobalamin A or B deficiency have a milder course [64]. Hörster et al. report that 46% of *MUT* patients experienced CKD with progression to KF [65] while only 9% of the CblA group experience KF. Recently, CKD has been reported as a long-term complication in PA [66]. In all age groups, cystatin C clearance identified 25/30 patients with an eGFR <90 ml/min/1,73 m^2^ while creatinine failed to identify them, underlining the importance to choose the appropriate read-out parameter in IMD patients. Glutaric aciduria type I (GA1) had been categorized as an exclusively cerebral organic aciduria. The I-IMD study population (*n* = 150 patients), however, revealed kidney disease in 25% of the adult patients [67]. No specific pattern could be determined (“CKD”); however, a GA1 mouse model revealed kidney tubular damage with thinned brush border membrane and altered mitochondrial morphology [68].

Two case reports discuss the uncommon occurrence of renal tubular acidosis in carnitine palmitoyltransferase 1 (CPT1) deficiency [69,70]; however, no follow-up data has been provided on this matter.

Finally, renal tubular acidosis has been reported in 15% of patients suffering with Vici syndrome, an EPG5-related disorder, which affects maturation of autophagosomes [71].

This section highlights the vital importance of kidney tubular function in promoting kidney health, while showcasing how monogenetic IMDs can impact specific segments of the kidney tubules.

## 6. Kidney cysts

Classical cystic kidney diseases are defined as ciliopathies and often associated with multi-organ involvement [72]. Cystic kidney disease has been reported as a characteristic feature in various monogenetic inborn errors of metabolism: Patients with disorders of fatty acid oxidation (multiple acyl CoA dehydrogenase deficiency (*ETFDH*) and electron transfer flavoprotein α/β subunit deficiency (*ETFA*/*ETFB*)), especially in the neonatal early onset forms, have presented with enlarged, polycystic kidneys. Antenatal oligohydramnios leading to Potter sequence has been reported [73–75].

Disorders of peroxisomal fatty acid oxidation (peroxisomal straight-chain acyl-CoA oxidase deficiency, D-bifunctional protein deficiency) and peroxisomal biosynthesis (peroxin deficiency 1, 2, 3, 6, 12, 13, 14B, 16, 19, 26) also frequently present with kidney cortex cysts. In D-bifunctional protein deficiency, 33% of investigated patients showed kidney cysts [76]. Severe clinical courses seem to have higher risk of cysts, while milder courses have been associated with calcium oxalate kidney stones [77].

Besides glomerular involvement (see above) kidney cysts are another characteristic finding in quite a number of CDGs. GANAB-CDG due to alpha glucosidase II deficiency has recently been linked to polycystic kidney (50% of reported patients) and liver disease [78]. ALG-9 CDG belongs to the group of N-glycosylation disorders. In 36% of reported patients, polycystic kidneys were detected. [79]. In a cohort of genetically unresolved cases with polycystic kidneys, 17% revealed to be due to ALG5-CDG caused by monoallelic pathogenic *ALG5* variants; 35% of the identified patients progressed to KF [80]. Some PMM2-CDG patients (see above) have been identified with polycystic kidneys [35]. Specifically, a promoter variant in *PMM2* leads uniformly to polycystic kidney disease and hyperinsulinemic hypoglycemia [81].

Kidney cysts include a relatively small group of IMDs comprising fatty acid oxidation, peroxisomal degradation and glycosylation disorders. However, if apparent, they are a quite characteristic feature of the respective diseases.

## 7. Kidney stones

Nephrolithiasis (or kidney stones) is a frequent finding in the population affecting approximately 10% of adults worldwide [82]. Medullary nephrocalcinosis (microscopic kidney interstitial crystal deposition) has been reported in some of these patients

Quite a few IMDs from different metabolic pathways present with such features: Cystinuria is caused by pathogenic variants in the *SLC3A1* and *SLC7A9* genes coding for a cystine transporter in PT cells which shuttles dibasic amino acids (cystine, arginine, lysine, ornithine) [83]. Cystinuria is the most common cause for monogenic kidney stones and is responsible for 1% of kidney stones in adults and 7% in children [84].

Cystinosis primarily leads to RFS (see above). However, kidney stones (12%) and nephrocalcinosis (33% mild; 20% severe) have been reported [85,86].

Disorders of tyrosine metabolism have also been linked to kidney deposits: Alkaptonuria is caused by pathogenic variants in the *HGD* leading to homogentisate 1,2-dioxygenase deficiency. Consequently, homogentisic acid cannot be properly degraded and leads to kidney stones and KF; 28% of patients have experienced an episode of nephrolithiasis [87,88]. In tyrosinemia, nephrocalcinosis was reported in 16% of reported children; kidney abnormalities persisted after liver transplantation [58].

Disorders of glyoxylate and oxalate metabolism also present with nephrocalcinosis and nephrolithiasis: Primary hyperoxaluria (PH) is characterized by hyperexcretion of oxalate, which leads to urolithiasis. Three subtypes (PH 1–3) are known. PH1, caused by pathogenic variants in the *AGXT* gene, leads to early symptoms in children. By the time of diagnosis (around age 7), 43% already have reached KF [89]. In PH2, caused by pathogenic variants in *GPDH*, 83% of the patients develop urolithiasis and 50% have CKD but are in general older [90]. PH3 is caused by pathogenic variants in the *HOGA* gene leading to repetitive kidney stones (calcium oxalate) in 93% of the patient collective and induces CKD [91]. Oxalate transporter deficiency coded by the *SLC26A1* gene also leads to nephrocalcinosis and urolithiasis. The contribution of pathogenic variants in *SLC26A1* gene which are causative for nephrolithiasis remain unclear [92]. Recently, *SLC26A1* variants have been linked to disturbed sulfate reabsorption in the kidney raising controversial discussions on the function of the transporter coded by the *SLC26A1* gene [93].

Also, pathogenic *OCRL* variants leading to Dent’s disease have been reported in the context of kidney precipitate formation [94].

Purine and pyrimidine disorders have a close association to kidney stone formation. Patients suffering from disorders of purine metabolism are especially at risk: Lesch-Nyhan syndrome is caused by pathogenic variants in the *HPRT* gene leading to excessively high uric acid concentrations. While neurological symptoms are very pronounced, about 9% of the patients experience urolithiasis [95]. Acute hyperuricemic nephropathy is frequently reported in about 2/3 of the patients [96]. Adenine phosphoribosyltransferase (APRT) deficiency leads to massive excretion of 2,8-dihydroxyadenine (DHA). DHA precipitates and induces formation of kidney stones. At the time of diagnosis, about 60% of the patients have developed kidney stones. If untreated (or not diagnosed), about 25% of the patients will develop KF in adulthood [97] [98]. Hereditary xanthinuria is caused by deficiency of the enzyme xanthine oxidase. Very low concentrations of uric acid are characteristic, while xanthine precipitates in high amounts and leads to kidney stones in about 50% of the patients [99]. Phosphoribosyl pyrophosphate synthetase (PRPS) superactivity is characterized by hyperuricosuria and hyperuricaemia. In the severe form (25% of patients), severe developmental delay, seizures and uric acid stone formation are described as main features [100]. Uridine monophosphate synthase (UMPS) deficiency, characterized by elevated excretion of orotic acid in urine, has been associated with crystalluria and kidney stone formation in some patients. However, megaloblastic anemia, neutropenia, developmental delay, and failure to thrive are more prominent features of this disease [101].

Nucleic acids are constantly degraded and processed to their purine nucleotides. The last step of purine degradations involves xanthine dehydrogenase producing uric acid. Molybdenum cofactor (MoCo) deficiency is caused by pathogenic variants in different genes (*MOCS1, MOCS2, GPHN, MOCOS*) and negatively affects xanthine dehydrogenase activity. Patients suffering from MoCo deficiency present with intractable seizures, axial hypotonia, and hyperekplexia; however, kidney stones add comorbidity to the disease and can be the presenting sign [102].

5-Oxoprolinuria linked to glutathione metabolism has a very heterogeneous clinical picture including kidney stone formation [103].

While TALDO deficiency primarily leads to tubular dysfunction (see above), kidney stones have been reported in 12% of patients [53].

Finally, FUT8-CDG is an ultra-rare condition caused by deficiency of the Golgi enzyme 1,6 fucosyltransferase. In the few cases reported, 2/3 of the patients developed nephrocalcinoses or kidney stones [104].

## 8. Malformations

Malformations of the kidneys account for 20–30% of detected abnormalities in the antenatal period; 30–50% lead to KF, underlining the importance for an early diagnosis and treatment [105]. Kidney anomalies have also been associated with IMD:

Patients with FBS, primarily associated with RFS (see above), classically present with nephromegaly due to glycogen storage [50,51]. Nephromegaly has also been described in patients with GSD type Ia/b [29].

Kidney hypoplasia and agenesis are a common feature of several enzymatic defects impacting sterol biosynthesis. The build-up of cholesterol is inhibited already early in embryogenesis: Smith-Lemli Opitz syndrome is caused by deficiency of 7-dehydrocholesterol reductase. The clinical spectrum is broad including characteristic craniofacial appearance, intellectual disability, 2–3 toe syndactyly, genital malformations, and multi-organ (heart, lung, gastrointestinal) involvement. Kidney anomalies are found in 25% of the patients. Fetal oligohydramnios sequence due to kidney agenesis has been detected in several cases [106]. X-linked chondrodysplasia punctata type 2 comprises skeletal abnormalities, scaling ichthyosis and cataracts. Hydronephrosis has been reported as a kidney complication [107]. Congenital Hemidysplasia with Ichthyosiform erythroderma and Limb Defects (CHILD) syndrome is a rare X-linked dominant ichthyotic disorder. Sterol biosynthesis is disturbed due to pathogenic variants in the *NSDHL* gene; kidney abnormalities have been described [108].

Nicotinamide adenine dinucleotide (NAD) is a cofactor needed for >400 intracellular reactions. In the embryonal stage, mammals need to synthesize NAD from tryptophan via kynurenine. 3-Hydroxykynureninase deficiency (*KYNU*) and 3-hydroxyanthranilic acid 3,4-dioxygenase (*HAAO*) are involved in the biosynthesis of NAD. Deficiency of either enzymes have been related to various anomalies of different organs including the kidney [109]. NAD synthetase 1 deficiency coded by the *NADSYN1* gene processes the last step of NAD synthase. Besides other organ anomalies, kidney embryogenic development can be disturbed in these patients [109].

Furthermore, fatty acid oxidation disorders with disturbed electron transfer FAD-dependent dehydrogenases as well as carnitine palmitoyltransferase II deficiency have been associated with kidney malformations and kidney cysts in particular [73–75,110].

Several mitochondrial diseases of different origin (e.g., respiratory chain defects, mitochondrial depletion syndromes, variants in mitochondrial/nuclear DNA, CoQ_10_ deficiencies) have been associated to kidney structural abnormalities including both kidney hypoplasia and dysplasia [111].

Cytochrome P450 oxidoreductase deficiency (PORD), a disorder of steroidogenesis, leads to cortisol deficiency, disorders of sex development and skeletal abnormalities. Kidney pelvic dilatation and vesicoureteral reflux are found in about 8% of affected patients [112].

Menkes disease, a disorder of copper metabolism, is caused by pathogenic variants in the *ATP7A* gene. Neurodegeneration, connective tissue abnormalities, and “kinky” hair are characteristic; 57% of the patients suffer from bladder diverticula as an anomaly of the urinary tract [113].

The origin of structural anomalies of the kidney in IMDs is thus variable, and their presence should draw clinical attention to a possible underlying IMD.

## 9. Differential diagnosis

**Fig. 2.**
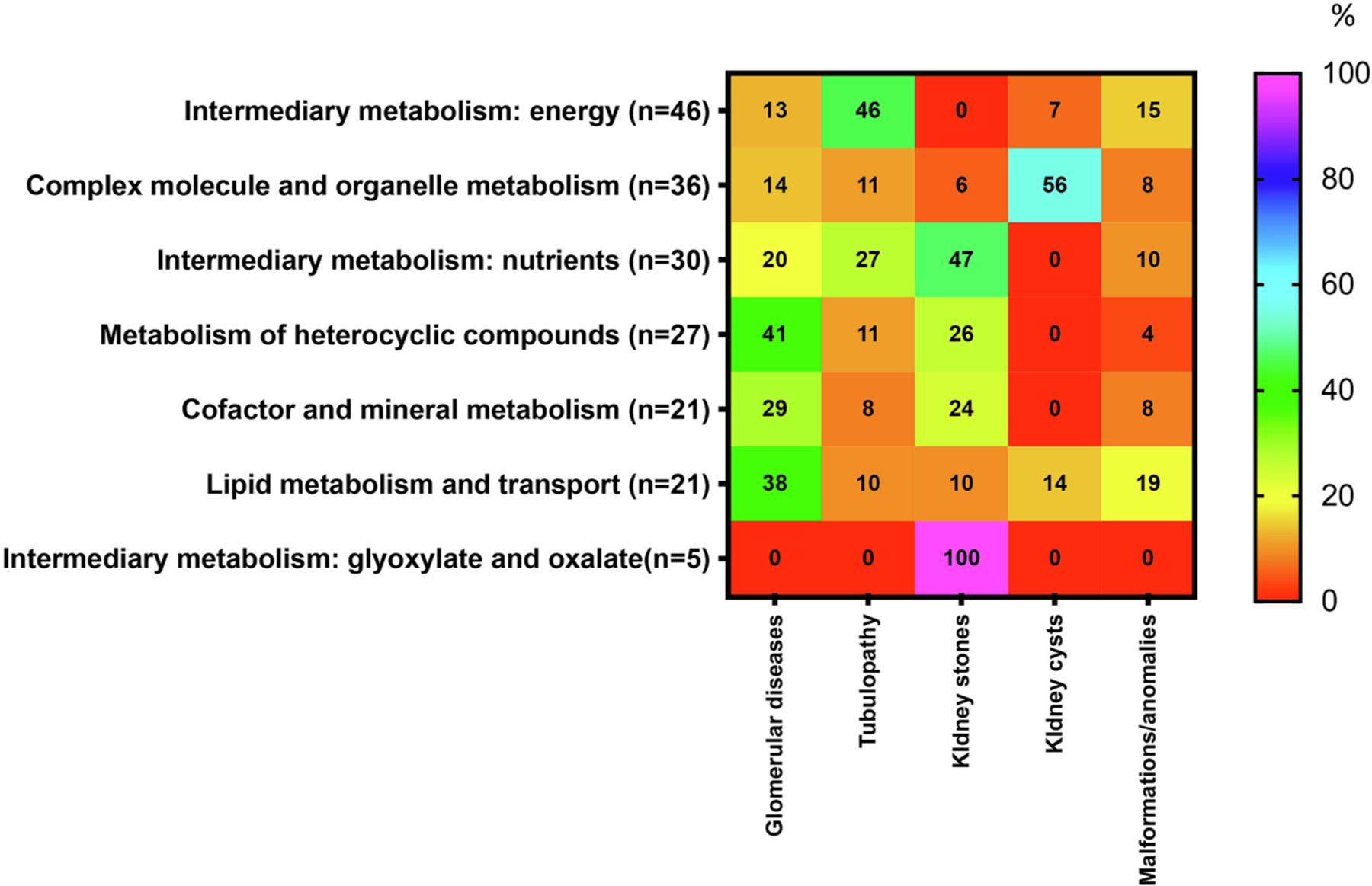
Occurrence (%) of symptoms associated with disorders presenting with kidney abnormalities in seven categories of IMDs (according to ICIMD). The percentages for renal abnormalities or dysfunction were calculated using as the denominator the total number of IMDs in each category presenting with any kidney characteristic. The heat scale ranges from red (0%) for diseases with no particular reported symptoms to violet (100%) for diseases with particular symptoms occurring more frequently within the disorders group. For the definition of six categories of seizure or epilepsy characteristics, see Supplemental Table 1. For interpretation of the references to colour in this figure legend, the reader is referred to the web version of this article. (For interpretation of the references to colour in this figure legend, the reader is referred to the web version of this article.)

Glomerular diseases are the most common kidney abnormality reported in 43/190 (23%) disorders, followed by tubulopathies in 41/190 (22%), kidney stones in 35/190 (18%), and kidney cysts in 26/190 (14%) (Fig. 2). Some of them are, however, specific for a particular disorder group, e.g., ‘kidney cysts’ in ‘Disorders of complex molecules and organelle metabolism’ and ‘Disorders of lipid metabolism and transport’ only. Of the signs and symptom in the group ‘Other’, most frequently reported among all disorders are ‘kidney failure, acute or chronic’ (51%), ‘kidney insufficiency’ (9%), ‘urinary infection’ (6%), ‘hematuria’ and ‘hydronephrosis’ (5%) each, and ‘kidney colic’ and ‘polyuria’ (4%) each. (Supplemental Table 1). Again, some of these symptoms are specific for some disorder subgroups, e.g., ‘hemolytic uremic syndrome’ in ‘Disorders of cobalamin metabolism’ or ‘congenital kidney anomalies’ in ‘Disorders of riboflavin metabolism’ and ‘Disorders of glycosylation’.

A list of laboratory investigations to aid in the diagnosis of the various listed IMDs is summarized in Table 1. A detailed list of laboratory tests is tabulated in the Supplemental Table 2. In disorders where metabolite testing is uninformative, diagnosis relies on molecular/genetic analysis as well as a thorough clinical history to identify characteristic presenting features. Importantly, patient stabilization during any episode of acute metabolic decompensation should be undertaken even before a specific diagnosis is made.

## 10. Conclusion

We provided a comprehensive list of 190 IMDs associated with renal manifestations and proposed a list of investigations to be performed based on the respiratory phenotypes as well as available treatment options. This represents the 14th issue in a series of educational summaries providing a comprehensive and updated list of metabolic differential diagnoses according to system involvement. The full list can be freely accessed at http://www.iembase.org/gamuts and will be curated and updated on a regular basis.

## Supplementary Material

1

2

## Figures and Tables

**Table 1:** Biochemical investigations in inherited metabolic diseases affecting kidney. B: blood, CSF: cerebrospinal fluid, DBS: dried blood spot, P: plasma, S: serum, U: urine.

Basic tests	Profiles	Special tests
Blood count	Amino acids (P,U)	Oxalate (P,U)
Sodium (P)	Organic acids (U)	Glycolate (P,U)
Potassium (P)	Acylcarnitines (DBS, P)	Glycerate (U)
Calcium (P)	Sialotransferins (S)	Copper (S,U)
ASAT/ALAT (P)	Sterols (P)	Ceruloplasmin (S)
CK (P)	Oligosaccharides (U)	Iron (S)
Lactate (P)	Mucopolysaccharides (U)	Manganese (B)
Glucose (P)	VLCFA (P)	Carnitine (P)
Ammonia (B)	Lipid panel (S)	Lysosomal Enzymes (S)
Coagulation factors	Guanidino compounds (U,P,CSF)	Vitamins (S)
Immunoglogulines	Polyols (P,U)	Interferon-alpha (CSF) DNA (Trio)

## Data Availability

Data will be made available on request.
